# Trace element fingerprinting of cockle (*Cerastoderma edule*) shells can reveal harvesting location in adjacent areas

**DOI:** 10.1038/srep11932

**Published:** 2015-07-07

**Authors:** Fernando Ricardo, Luciana Génio, Miguel Costa Leal, Rui Albuquerque, Henrique Queiroga, Rui Rosa, Ricardo Calado

**Affiliations:** 1 Departamento de Biologia & CESAM, Universidade de Aveiro, Campus Universitário de Santiago, 3810-193 Aveiro, Portugal; 2 MARE – Marine and Environmental Sciences Centre, Faculdade de Ciências da Universidade de Lisboa, Campo Grande, 1749-016 Lisboa, Portugal

## Abstract

Determining seafood geographic origin is critical for controlling its quality and safeguarding the interest of consumers. Here, we use trace element fingerprinting (TEF) of bivalve shells to discriminate the geographic origin of specimens. Barium (Ba), manganese (Mn), magnesium (Mg), strontium (Sr) and lead (Pb) were quantified in cockle shells (*Cerastoderma edule*) captured with two fishing methods (by hand and by hand-raking) and from five adjacent fishing locations within an estuarine system (Ria de Aveiro, Portugal). Results suggest no differences in TEF of cockle shells captured by hand or by hand-raking, thus confirming that metal rakes do not act as a potential source of metal contamination that could somehow bias TEF results. In contrast, significant differences were recorded among locations for all trace elements analysed. A Canonical Analysis of Principal Coordinates (CAP) revealed that 92% of the samples could be successfully classified according to their fishing location using TEF. We show that TEF can be an accurate, fast and reliable method to determine the geographic origin of bivalves, even among locations separated less than 1 km apart within the same estuarine system. Nonetheless, follow up studies are needed to determine if TEF can reliably discriminate between bivalves originating from different ecosystems.

The global production of bivalves (e.g. cockles, clams, mussels and oysters) has notably increased since the 1990s, reaching over 16 million tons in 2012^1^. Bivalves are filter-feeders and are able to retain, accumulate and concentrate pathogens (e.g. *Salmonella* and *Vibrio*) known to cause foodborne infections worldwide^2^. Consequently, this high trade volume of bivalves, combined with its raw or lightly cooked consumption, represents a potential risk to global human health^3^. It is, therefore, acknowledged that supply chains trading live bivalves presently rank food safety issues at the top of their priorities^4^.

Council Regulation 853/2004 and 854/2004^5^,^6^ set the microbiological safety standards for bivalves destined for human consumption in the European Union (EU). Briefly, EU fishing/production areas for bivalves are ranked as A, B or C according to the levels of *Escherichia coli* present in the flesh and intra-valvular liquid of live specimens, with this feature determining whether they are suitable for human consumption immediately after harvesting or need to undergo depuration procedures^5^,^6^,^7^,^8^. In most EU countries, the fishing/production of bivalves is centred in estuaries and coastal lagoons, being common to have different classifications within the same aquatic system. Therefore, tracing the origin of traded bivalves to their specific fishing/production area, even within the same aquatic system, is paramount to ensure public food safety^9^. As a consequence, the EU developed specific requirements for seafood traceability. Particularly, article 58 of EC 1224/2009 requires that “*all lots of fisheries and aquaculture products shall be traceable at all stages of production, processing and distribution, from catching or harvesting to retail stage*”. More recently, the European regulation (EC) No 1379/2013 “*on the common organization of the markets in fishery and aquaculture products”* further contributes to the implementation of seafood traceability and requires that the category of fishing gear or production method (i.e. caught or farmed) is provided together with geographic detail of the catch area. However, this information is not always available to end consumers and is prone to fraudulent use (e.g. mislabelling of place of origin). Consequently, even conscientious buyers aware of the potential hazards associated with the consumption of bivalves may not be able to securely purchase this highly-prized seafood. It is critical to develop and validate reliable techniques that allow competent authorities to trace the origin of traded bivalves to ultimately fight fraud and prevent notable risks to public health^10^.

The taxonomic identification of bivalves and the traceability of their fishing location are often difficult to achieve, as bivalves are commonly processed after collection (e.g. precooked, canned). Most studies have relied on molecular techniques, including PCR^11^,^12^, FINS^13^,^14^ and DNA barcoding^15^,^16^, for species identification. Molecular tools, particularly microsatellites^17^,^18^, as well as biochemical methods, such as fatty acids^19^,^20^,^21^ and stable isotopes^21^,^22^,^23^, have also been used to assess geographical origin of bivalves. Trace element fingerprinting (TEF) of bivalve mineral structures may also be useful to distinguish populations or stocks^24^,^25^,^26^. Trace elements are influenced by environmental features of each ecosystem^27^ and are recorded in hard structures (e.g. shells, statolith and otoliths) of marine organisms^28^. Several trace elements are found in a wide range of marine species^29^, with the most common being aluminium (Al), barium (Ba), calcium (Ca), cobalt (Co), chromium (Cr), copper (Cu), magnesium (Mg), manganese (Mn), lead (Pb), zinc (Zn) strontium (Sr) and uranium (U). TEF of sea snails larvae^28^ and shells from bivalve larvae^30^ and adults^26^ have been successfully used to distinguish specimens from geographically close populations (20–50 km). However, it remains unclear whether this geochemical approach has enough resolution to discriminate specimens from adjacent areas (<1 km apart) within the same aquatic system.

The present study aimed to validate TEF of shells from fresh bivalves as a proxy to discriminate the origin of specimens collected from adjacent areas of the same estuarine system. It is important to highlight, that unlike previous studies on TEF that use laser ablation of a small part of the larval or early juvenile shells of bivalves^28^,^30^, the present study uses the whole shell of adult bivalves. The rationale for using this approach was to somehow minimize the temporal variability of TEF in the shells of adult specimens. We used cockle (*Cerastoderma edule*) as a model species due to its economic importance as a fishery resource^31^, with the coastal lagoon Ria de Aveiro (Portugal) being selected as the collection site due to its diverse tidal system and important role in Portuguese bivalve fisheries^31^. Once cockles are usually fished by hand or hand-raking, this study also aimed to test if the use of metal rakes could induce some type of metal contamination and be a source of bias for TEF. The following hypotheses were tested i) TEF of *C. edule* shell does not differ with fishing method (i.e. hand-raking *vs.* by hand), and ii) TEF of *C. edule* shell is similar among different locations within the same coastal lagoon.

## Material and Methods

### Study area and cockle collection

**Figure 1:**
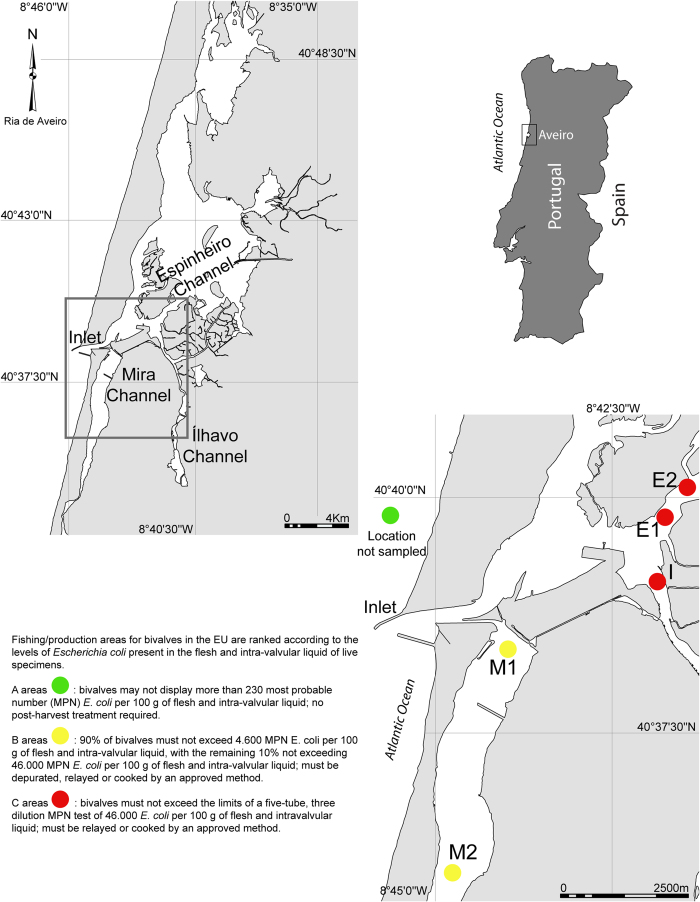
Sampling locations of *Cerastoderma edule* in Ria de Aveiro, Portugal: Mira Channel (M1/M1A: 40°38'26.30"N, 8°43'58.90"W and M2: 40°35'58.30"N, 8°44'47.80"W), Ílhavo Channel (I: 40°38'35.40"N, 8°41'35.40"W) and Espinheiro Channel (E1: 40°39'48.50"N, 8°41'45.03"W and E2: 40°40'2.72"N, 8°41'26.08"W). The map was created using the software ArcGIS v9.2.

*Cerastoderma edule* with a shell length > 25 mm (i.e. commercial size) (likely displaying an age of 3+ years; the species lifespan may be up to 6 years^32^) were collected during June 2013 in five different locations of Ria de Aveiro distributed among Mira (M1 and M2), Espinheiro (E1 and E2) and Ílhavo (I) Channels (Fig. 1). All locations play an important role on the fishery of *C. edule* in Ria de Aveiro, which usually exceeds 1000 tons per year in this region^31^. Two fishing methods were used to collect twenty specimens of *C. edule* at M1: ten by hand-raking and ten by hand (*n* = 10 *2). Subsequently, ten specimens were collected by hand on the other locations: M2, E1, E2 and I (Fig. 1). All samples were stored in aseptic bags kept refrigerated during sampling and brought to the laboratory and frozen at −20 °C for later processing.

### Shell preparation

Volumetric polyethylene material and micropipettes with plastic tips were used to prepare collected shells for trace elements analysis^33^. Plastic bottles, ceramic coated blades and tweezers kept in 2–5% solution of DECON 90 over 2 h were washed with running water, immersed in 10% of HNO_3_ for 24 h, washed with Milli - Q (Millipore) water and dried in a laminar flow hood. The preparation for ICP-MS analysis was performed in a class 100 (ISO class 5) clean room. The valves were separated and the organic tissues were removed using ceramic coated blades and tweezers. The right valve was transferred to a previously acid-washed plastic bottle and the left valve discarded.

Samples were soaked in 20 mL high-purity H_2_O_2_ (30% w/v) (AnalaR NORMAPUR, VWR Scientific Products) overnight (14–16 h) to remove organic matter from the shell including the periostracum. After organic matter removal, the valve was rinsed in Milli – Q (Millipore) water three times. Digestion of entire valves was performed with addition of 20 mL of high-purity concentrated (70% w/v) HNO_3_ (Trace metals; Sigma-Aldrich). To avoid having Ca masking the concentrations of the remaining elements^34^,^35^, the resulting solution was diluted with Milli – Q (Millipore) water to a final acid concentration of 2% HNO_3_.

### ICP-MS analysis

Samples were analysed for total aluminium (Al), barium (Ba), calcium (Ca), cadmium (Cd), copper (Cu), magnesium (Mg), manganese (Mn), lead (Pb), strontium (Sr) and zinc (Zn) by an accredited laboratory at the University of Aveiro (Portugal). The concentrations of ^27^Al, ^137^Ba, ^111^Cd, ^65^Cu, ^55^Mn and ^66^Zn were determined through inductively coupled plasma mass spectrometry (ICP-MS), on a Thermo ICP-MS X-Series equipped with a auto sampler CETAX ASX-510, Peltier Nebulizing Camera Burgener nebulizer, nickel cones and the CeO+/Ce+ ratio was optimized at <2%. The concentrations of ^48^Ca, ^24^Mg and ^88^Sr were determined by inductively coupled plasma optical emission spectrometry (ICP-OES) on a ICP-OES Jobin Yvon Activa M equipped with auto sampler JY-AS500 and Burgener Mira Mist nebulizer.

### Statistical analysis

Concentrations of trace elements of the shells were standardized to Ca and all data analyses were carried out on the element ratios (X: ^48^Ca)^25^,^30^,^36^. To assess if fishing method significantly affected TEF, a resemblance matrix based on the normalized Euclidean distance was calculated^37^ for a one-way analysis of similarity (ANOSIM)^38^, which calculates a global R statistic that assesses the differences in variability between groups when compared to within groups and checks for the significance of R using permutation tests^38^. Differences among fishing locations for each elemental ratio were assessed using a one-way analysis of variance (ANOVA), and Tukey’s HSD pairwise comparisons when significant differences were observed (*p* < 0.05). Similarity percentages (SIMPER) were calculated to quantify the contribution of each trace element to the dissimilarities recorded among locations. Only trace elements that cumulatively contributed up to 80% of the dissimilarities recorded were selected^39^. A Canonical Analysis of Principal Coordinates (CAP)^39^ was performed to test if TEF could be used to predict the fishing location of collected specimens. CAP is a constrained ordination tool that discriminates locations defined *a priori* and determines the level of misclassification among sampling locations. Appropriate axis (*m*) was applied by maximizing the leave-one-out allocation success (*m* = 5)^40^. This approach tests how well locations were discriminated using CAP. To quantify the effect of each trace element to potential differences recorded among locations, Spearman correlation were calculated for all trace elements and the CAP axes. Only the trace elements with a correlation coefficient (*r*) > 0.30 were considered. ANOVAs were performed using GraphPad Prism 6 (GraphPad Software. Inc., San Diego, CA, USA), and multivariate analyses were performed using PRIMER v6 with the add-on PERMANOVA + .

## Results

**Figure 2:**
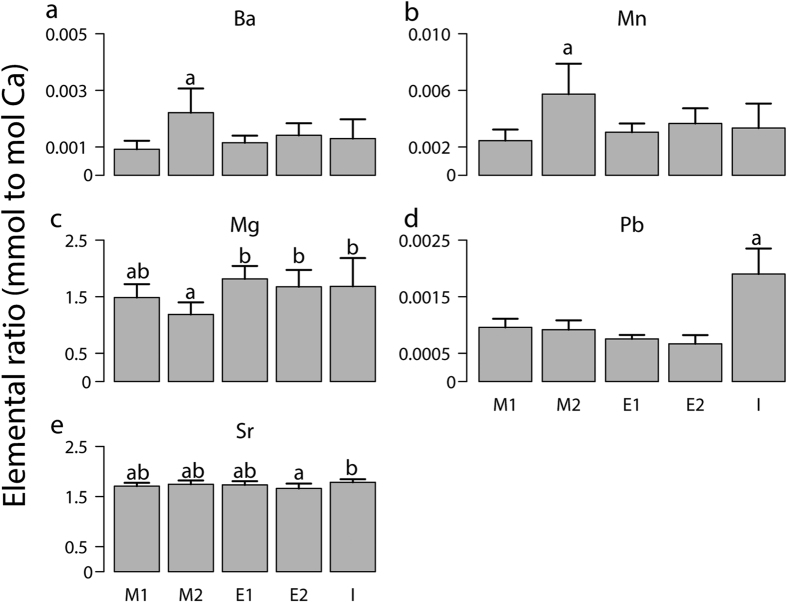
Ratios of trace elements to Calcium (Ca) concentrations (mmol to mol) (average ± SD; *n* = 10) of *Cerastoderma edule* shells from five locations within Mira (M1 and M2), Espinheiro (E1 and E2) and Ílhavo (I) Channels in Ria de Aveiro (Portugal). Significant differences (*p* < 0.05) among different locations are noted with different letters.

Five trace elements (^137^Ba, ^24^Mg, ^55^Mn, ^207^Pb and ^88^Sr) were detected in *C. edule* shells from Ria de Aveiro, with Mg and Sr denoting the highest ratios to Ca (Fig. 2). While no differences between specimens collected by hand or by hand-raking were detected (ANOSIM, *p* *=* 0.268, *R* = 0.025), significant differences among locations were observed for each trace element ratio (Fig. 2; one-way ANOVA, *p* < 0.05 for all trace elements; Table 1, summarizes ANOSIM results). The ratios of Mn and Ba were significantly higher at location M2 (*p* *=* 0.0001 and 0.0001, respectively). In contrast, the Mg ratio was lowest for *C. edule* shells from M2 (*p* *=* 0.0004). The Pb ratio was only significantly higher (*p* *=* 0.0001) at location I, whereas the Sr ratio was also higher at this location but only significantly different from shells collected at E2.

Pairwise comparisons revealed significant differences among locations, apart from those within the Espinheiro Channel, i.e. E1 and E2 (ANOSIM, *p* = 0.059, R = 0.123). SIMPER analysis showed that the dissimilarity among locations was associated to five elemental ratios: Mg, Sr, Pb, Ba and Mn. (Table 2). Mg and Sr were always among the elements that most contributed for the variability between location M1 and locations from Espinheiro Channel (E1 and E2). Mg and Sr varied significantly between M1 and Espinheiro (p = 0.0001 and 0.021, respectively) and SIMPER revealed that these elements explained more than 55% of the differences recorded between these locations (Table 2). Specimens from location I were significantly different from other areas due to their concentrations of Pb (Fig. 2). SIMPER analysis revealed that Pb alone accounted for 28 to 53% of all differences recorded between location I and all other locations (Table 2). Ba and Mn together contributed for more than 43% of the differences recorded among specimens collected in M2 and other locations.

**Figure 3:**
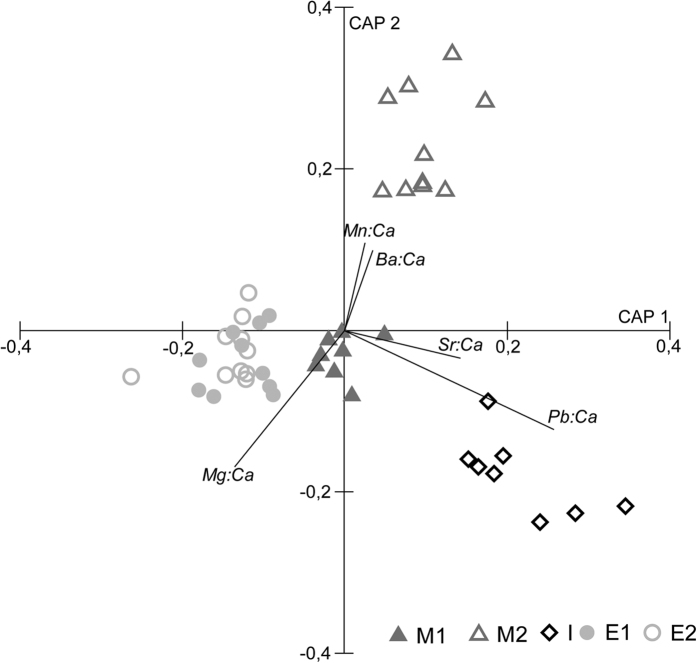
Canonical analysis of principal coordinates (CAP) based on Euclidean distances matrices of normalized elemental ratios, with axes drawn to maximize discrimination among assemblage types. Vector overlay Spearman correlations of trace elements composition with canonical axes are shown if |*r*| > 0.30.

TEF differences among locations were strong enough to accurately assign collected specimens to their fishing location. The leave-one-out procedure resulted in an average CAP classification of 92% (Table 3), i.e. 92% of the specimens were correctly assigned to their origin. Locations M1, M2 and I had the highest percentage of correct classification (100%), whereas two replicates from E1 and E2 were misclassified, which led to 80% correct classifications. Vector overlay of Spearman correlations of TEF with CAP axes are shown in Fig. 3. Vectors of Ba and Mn ratios were positively correlated with samples from location M2, Mg ratio with areas E1 and E2, and Pb and Sr ratios associated with samples from location I (Fig. 3). *C. edule* from area M1 were not associated with a particular trace element.

## Discussion

In general, adult bivalves display a reduced locomotor ability, being their aragonitic shells potential biogenic archives of marine ecosystems environmental fingerprints^41^. This feature prompted the use of trace elements of bivalve shells to assess their geographic origin. TEF has been successfully used to geographically distinguish populations of blue mussel *M. edulis*^26^, black mussel *M. galloprovincialis* and California sea mussel *M. californianus*^29^,^30^, soft shell clam *Mya arenaria*^36^ and Olympia oyster *Ostrea lurida*^42^. This geochemical tool also allowed to distinguish juveniles of green-lipped mussel (*Perna canaliculus*) ~13 km apart^43^, and to record differences in scallop shells (*Argopecten irradians*) within a small bay (~10 km^2^)^44^. The present study shows, for the first time, that TEF of bivalve shells can be used to assign the origin location of bivalves with a resolution <1 km.

While Mg and Sr ratios were relatively higher than Ba and Mn (Fig. 2), the latter ratios were among the most important to differentiate locations (Table 2). The presence of Ba and Mn with elevated concentration, as observed in M2, have been already reported for *Isognomon ephippium*^45^, *Mercenaria mercenaria*, *Spisula solidissima*^46^ and *M. edulis*^47^. Such high concentration in Ba and Mn are usually associated with freshwater inputs and nutrient runoff to estuarine systems, which ultimately causes phytoplankton blooms, particularly diatoms^46^,^47^,^48^,^49^. It is possible that the environmental conditions at M2, which is located more upstream and has stronger riverine input, causes diatom blooms more often than the conditions observed at others locations such as M1, which is located near the inlet^50^. Ba and Mn end up in bivalves’ tissue and shell as a consequence of the ingestion of Ba and Mn-rich particles associated with such diatom blooms. Heavy metals are also incorporated in calcite and aragonite shells of bivalves^51^,^52^. The high levels of Pb in the shells from location I are likely associated with anthropogenic impacts, particularly acute pollution from boats using leaded gasoline. Note that location I is relatively close to the commercial harbour of Aveiro.

Cockle shells from each location displayed a different TEF, with the exception of stations E1 and E2 that showed no statistical differences between each other (Table 1). Nevertheless, CAP results showed a success of 80–100% to identify the origin of cockles collected from Ria de Aveiro (Table 3). The important to highlight that the misclassifications were solely associated with locations E1 and E2. It is the potential of TEF for geographical traceability purposes as we were able to identify the origin of cockles using this statistical tool (CAP) in, at least, 80% of the cases. Nevertheless, the average 92% correct classification is still higher than results by Sorte *et al.*^26^ for the blue mussel *M. edulis* in the Gulf of Maine (68%) and by Becker *et al.*^30^ for the congeners mussels *M. californianus* and *M. galloprovinciallis* in Southern California, USA (56%). The latter and other studies^26^,^29^,^30^,^42^,^43^ have also shown that Ba, Mn, Mg, Pb and Sr play an important role discriminating specimens among areas, as observed here through the magnitude of the vectors of the standardized discriminant functions (Fig. 3).

The chemical nature of the trace elements deposited over time in bivalves is determined by metabolic efficiency and environmental conditions^53^. As this study was conducted in Ria de Aveiro, which is a highly dynamic tidal-system with notable spatial variability in environmental conditions^54^, it is likely that different fingerprints are associated with contrasting environmental conditions recorded at each channel (Fig. 1). The spatial variability here recorded for TEF of cockle shells thus stresses the potential of this method to validate screening for fraudulent use of origin certification. However, temporal variability in environmental conditions may also change TEF and interfere with this traceability tool. Indeed, it has already been shown that seasonal and annual variation may change the TEF of bivalves and other biogenic carbonate structures such as fish otoliths^29^,^30^,^55^. In opposite, Carré *et al.*^56^ showed that environmental changes have minor influence on Sr, Ba, Mg and Mn concentration in shell aragonite of the marine bivalve species *Mesodesma donacium* and *Chione subrugosa*. This study aimed to validate a tool for origin certification of bivalves and not to study the temporal variability of TEF. Consequently, we used cockles with similar size and, therefore, similar age, in order to minimize any bias associated with potential differences in the age of selected specimens. The analyses performed in this study used the whole shell and, consequently, averaged the present and the past elemental fingerprints of cockles. While this approach may have the TEF over multiple years, notable differences among sites were still recorded (Table 1), which emphasizes the robustness of this method for geographical traceability purposes. However, if legal authorities aiming to fight the fraudulent mislabelling of origin location want to minimize this potential temporal bias associated with the analysis of the whole shell, they may rather monitor the elemental fingerprint of the outer margins of bivalve shells from each fishing location or, those more prone to fraud, as these will reflect the most recent elemental fingerprints from the location where they were collected. By comparing the fingerprint of the investigated shells with monitoring data and/or samples from the different sites in the same season, legal authorities may minimize the effect of temporal variability and, ultimately, use of this tool to expose fraudulent situations.

Although TEF fails to detect differences associated with fishing method, this information would be potentially relevant for legal authorities to manage bivalve trade, from fishing to the end consumer, as fishermen using hand-raking usually collect larger volumes of bivalves. The effect of environmental conditions on the TEF of bivalves occurs within a relatively long time frame, i.e. within weeks or months^57^, which likely explains the lack of differences associated with fishing methods. The effect of fishing method on TEF, if any, would probably occur within a very small time frame as fishing duration usually takes less than one hour.

Most traceability tools have been focused on issues associated with species mislabeling^13^,^58^,^59^ or with identification of geographical origin of specimens separated by distances higher than 20 km^26^,^30^. However this study shows, for the first time, that TEF can be a fast, reliable and accurate method that may be used for origin certification of bivalves collected from locations less than 1 km apart. While this is probably associated with the high environmental variability observed within Ria de Aveiro, it is still unknown if TEF is a reliable tool to accurately identify the origin of bivalves collected from different ecosystems with similarly high variability. Follow-up studies are already being developed to clarify if TEF can be used to discriminate between bivalve shells from specimens originating from distinct ecosystems (from tens to hundreds of km apart). An additional benefit of TEF is that there is no post-harvesting shift and/or degradation associated with bacterial action as recorded for biochemical and molecular methods. The present approach may also play a relevant role on the conservation and management of cockle populations being exploited, namely in the fight against illegal/unreported fishing.

## Additional Information

**How to cite this article**: Ricardo, F. *et al.* Trace element fingerprinting of cockle (*Cerastoderma edule*) shells can reveal harvesting location in adjacent areas. *Sci. Rep.* **5**, 11932; doi: 10.1038/srep11932 (2015).

## Figures and Tables

**Table 1:** Similarity (ANOSIM) between trace elements fingerprinting of *Cerastoderma edule shell* from five locations within Mira (M1 and M2), Espinheiro (E1 and E2) and Ílhavo (I) Channels in Ria de Aveiro (Portugal).

Locations	*R*	*p*
M1 vs M2	0.503	0.002
M1 vs E1	0.238	0.025
M1 vs E2	0.202	0.028
M1 vs I	0.401	0.002
M2 vs E1	0.641	0.001
M2 vs E2	0.428	0.001
M2 vs I	0.563	0.001
E1 vs E2	0.123	0.059
E1 vs I	0.673	0.001
E2 vs I	0.643	0.001

**Table 2:** Similarity percentage analysis (SIMPER) identifying the elements contributing to the differences recorded in the shell of *Cerastoderma edule* from five locations within Mira (M1 and M2), Espinheiro (E1 and E2) and Ílhavo (I) channels in Ria de Aveiro, Portugal (Ind – individual; Cum – cumulative).

M1 vs M2			M1 vs E1			M1 vs E2			M1 vs I			M2 vs E1		
Element	Ind (%)	Cum (%)	Element	Ind (%)	Cum (%)	Element	Ind (%)	Cum (%)	Element	Ind (%)	Cum (%)	Element	Ind (%)	Cum (%)
Ba	38.52	38.52	Mg	38.32	38.32	Sr	35.57	35.57	Pb	41.50	41.50	Ba	29.75	29.75
Mn	38.47	76.99	Sr	34.08	72.40	Mg	20.80	56.37	Mg	16.69	61.19	Mn	29.56	59.31
Sr	11.31	88.31	Mn	10.16	82.56	Ba	17.35	73.72	Sr	16.24	77.42	Mg	27.61	86.93
						Mn	16.86	90.58	Ba	11.41	88.84			
**M2 vs E2**			**M2 vs I**			**E1 vs E2**			**E1 vs I**			**E2 vs I**		
**Element**	**Ind (%)**	**Cum (%)**	**Element**	**Ind (%)**	**Cum (%)**	**Element**	**Ind (%)**	**Cum (%)**	**Element**	**Ind (%)**	**Cum (%)**	**Element**	**Ind (%)**	**Cum (%)**
Mn	25.26	25.26	Pb	28.01	28.01	Sr	52.55	52.55	Pb	53.86	53.86	Pb	46.98	46.98
Ba	24.96	50.22	Mn	22.23	50.25	Mg	21.40	73.94	Mg	17.12	70.99	Sr	24.10	71.08
Sr	24.18	74.40	Ba	21.66	71.91	Ba	11.91	85.85	Sr	12.62	83.60	Mg	13.73	84.81
Mg	21.73	96.13	Mg	20.40	92.31									

**Table 3:** Classification success of cross-validation for cockle *Cerastoderma edule* based on trace elemental composition in the shell from five locations within Mira (M1 and M2), Espinheiro (E1 and E2) and Ílhavo (I) Channels in Ria de Aveiro, Portugal.

	Predicted Locations					Total per location	% correct (location)
	M1	M2	E1	E2	I		
**Original Area**							
**M1**	10					10	100
**M2**		10				10	100
**E1**			8	2		10	80
**E2**			2	8		10	80
**I**					10	10	100
**Average classification success**							92
